# Hyperbaric oxygen therapy for treatment of children with autism: a systematic review of randomized trials

**DOI:** 10.1186/2045-9912-2-13

**Published:** 2012-05-11

**Authors:** Ahmad Ghanizadeh

**Affiliations:** 1 Research Center for Psychiatry and Behavioral Sciences, School of Medicine, Shiraz University of Medical Sciences, Shiraz, Iran; 2 Department of Psychiatry, School of Medicine, Shiraz University of Medical Sciences, Shiraz, Iran

**Keywords:** Autism, Hyperbaric, Oxygen, Treatment, Management, Systematic, Review

## Abstract

There is a controversy about the efficacy of hyperbaric oxygen (HBO) therapy for the treatment of autism. This study systematically reviews the current evidences for treating of autism with HBO therapy. According to PRISMA guidelines for a systematic review, the databases of MEDLINE/Pubmed, Google Scholar, and Randomised Controlled Trials in Hyperbaric Medicine were electronically searched. In addition, medical subject heading terms and text words for hyperbaric oxygen therapy and autism were used. The main inclusion criteria were published studies which reported the original data from the trials conducted on the patients with autism and assessed outcomes with a valid and reliable instrument. A quality assessment was also conducted. The electronically search resulted in 18 title of publications. Two studies were randomized, double-blind, controlled-clinical trials. While some uncontrolled and controlled studies suggested that HBO therapy is effective for the treatment of autism, these promising effects are not replicated. Therefore, sham-controlled studies with rigorous methodology are required to be conducted in order to provide scientific evidence-based HBO therapy for autism treatment.

## Introduction

Hyperbaric oxygen therapy (HBO) is suggested for treating some medical problems, such as air or gas embolism, carbon monoxide poisoning, intracranial abscess, and radiation injury; however, its mechanism of action is not clear [1]. It is suggested that HBO increases the production of reactive oxygen species [2]. Moreover, HBO is a safe intervention [1] and middle ear barotraumas is one of its common adverse effect [3].

Autism is a complex neuro-developmental disorder with an increasing prevalence which is characterized by three main symptoms, including impairments in socialization and communication, restricted interests, and repeated behaviors. Meanwhile, there is no curative treatment for autism. Moreover, there are only two Food and Drug Administration (FDA) approved medications, including risperidone and aripiprazole for managing its symptoms [4]. Therefore, there is an urgent need to provide alternative therapeutic approaches for autism. In recent years, HBO is investigated as an alternative treatment for autism. Early uncontrolled studies reported the efficacy of HBO therapy. However, the results of later controlled-studies are controversial.

There are many reports about the possible role of neuro-inflammation in autism [5-9]. This neur-inflammation can be a possible target for the treatment of some cases with autism [10,11]. Besides, the regional cerebral blood flow is decreased in the bilateral frontal lobe, temporal, limbic system, and basal ganglias in autism spectrum disorders [12]. Moreover, it is proposed that HBO may improve the cerebral hypoperfusion and decrease brain inflammation as well as oxidative stress in autism [13,14]. On the contrary to some expectations, HBO therapy does not exacerbate the increased oxidative stress in autism [6,15]. Moreover, it does not affect plasma oxidized glutathione level. However, HBO therapy decreased C-reactive protein (CRP) level in a fasting blood sample [15]. Also, plasma concentration of some interleukins (IL), such as IL-1β, IL-1RA, IL-5, IL-8, IL-12(p70), IL-13, and IL-17 in the children with autism spectrum disorders is higher than that of the controls [16]. Nevertheless, HBO therapy did not affect the level of cytokines [17].

The present study is a systematic review of the current literature regarding the efficacy of HBO therapy for treating children with autism. There are two objectives: 1) assessment of the effects of HBO therapy for the treatment of autism, and 2) reporting adverse effects of HBO therapy in these children and adolescents.

## Method

### Literature search

This descriptive systematic review was conducted using the PRISMA (Preferred Reporting Items for Systematic reviews and Meta-Analyses) guidelines for searching the literature and reporting the results [18]. The author reviewed the titles and abstracts of the retrieved articles in order to assess if they met the established inclusion criteria.

Types of studies: The current study aimed to review all non-randomized and randomized clinical trials concerning the use of HBO for the treatment of autism symptoms. MEDLINE/Pubmed, Google Scholar, and the Database of Randomised Controlled Trials in Hyperbaric Medicine (DORCTIHM), which is a specifically targeted database of clinical evidence in the field of HBO http://www.hboevidence.com, were electronically searched. In addition, medical subject heading terms and text words for 'hyperbaric oxygen therapy' AND 'autism'; as well as 'hyperbaric oxygen therapy' and 'pervasive developmental disorder' were used. These databases were searched from their starting date to January 2012. Moreover, the references of all the included papers were searched.. Language was not considered as an exclusion criterion. The Diagnostic and Statistical Manual of Mental Disorders, 4th Edition, Text Revision, (DSM-IV-TR) was considered for autism diagnosis.

#### Types of participants

Participants of any age from both genders with autism spectrum disorders were included in the present study.

#### Types of interventions

Trials comparing the beneficial and harmful effects of HBO therapy with or without adjuvant pharmacotherapy were included. HBO protocol type was not considered as an exclusion criterion.

#### Types of outcome measures

The primary outcome measures assessed the outcomes with a valid and reliable instrument. Besides, the secondary outcome measures evaluated any reported adverse events of HBO therapy.

### Data extraction and validity scoring

**Figure 1:**
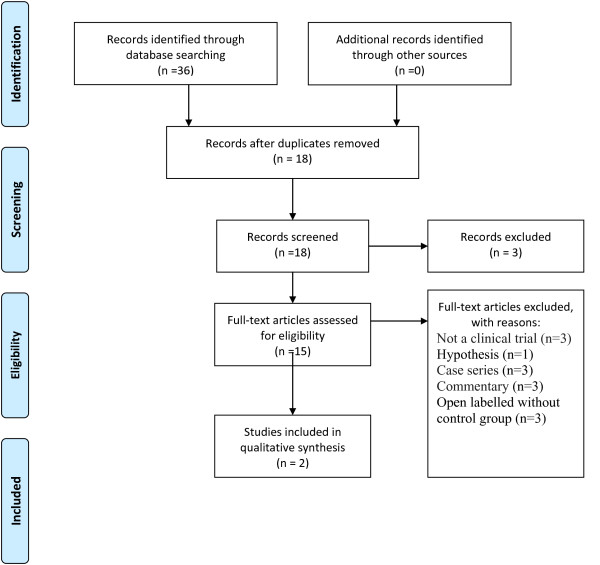
**Flowchart of PRISMA trial selection process**.

Both the titles and the abstracts were evaluated for inclusion based on the participants, the design of trial, intervention, and outcomes assessment. The articles which did not meet the inclusion criteria were excluded from the study. A summary of the flow of information is displayed in Figure 1. The modified four-item Oxford scale, whose score ranges from 0 to 7, (cited by [19,20]) was used in order to assess the methodological validity of the articles (Table 1). Also the information about the specific diagnosis, HBO therapy and its duration and protocols, autism symptoms as outcome, and secondary outcomes (adverse effects) were collected.

**Table 1:** Validity scoring of the trials

Study	Randomization*(0 to 2)*	Concealment of allocation (0 to 1)	Double-blinding*(0 to 2)*	complete reporting of data*(0 to 2)*	Total validity scoring
Granpeesheh [21]	*2*	*1*	*2*	*1*	*6*
Rossignol [22]	*2*	*1*	*2*	*1.5*	*6.5*
Rossignol [15]	*0*	*0*	*0*	*2*	*2*
Jepson [23]	*0*	*0*	*0*	*2*	*2*
Bent [17]	*0*	*0*	*0*	*2*	*2*

## Results

### Trial flow

In this study, a total of thirty six abstracts were identified. The initial review of the records showed that 18 articles were duplicated and three ones were irrelevant (Figure 1). Also, 15 full text articles were retrieved for additional information. Nevertheless, no unpublished data were retrieved, and no animal studies were found. It must be noted that all the retrieved papers were in English.

Only 2 studies were randomized, double-blind, controlled-clinical trials [21,22]. The retrieved published documents (articles) included a hypothesis [13], 3 case series [14,24,25], and 3 commentaries [26-28]. In addition, 3 articles were not clinical trials [29-31], and 3 studies were open labeled without any control groups [15,17,23].

### Primary outcome - efficacy

Overall, two published trials including a total of 89 participants met the inclusion criteria (Table 2). These two articles were a high quality. Moreover, both studies delivered HBO therapy at 24% oxygen and 1.3 atmospheric pressure to the treatment groups [21,22].

**Table 2:** Summary of included studies

References	Methods	Participants	Interventions	Measurements	Outcome and adverse effects
[21]	Multicenter, randomized, double-blind, controlled trial.	62 children with autism, ages 2-7 years old (mean 4.92 +/- 1.21), control group (n = 29) treatment group (n = 33)	40 hourly treatments. treatment group: hyperbaric treatment at 1.3 atmosphere (atm) and 24% oxygen, control group: slightly pressurized room air at 1.03 atm and 21% oxygen	Clinical Global Impression (CGI) scale, Aberrant Behavior Checklist (ABC) Autism Treatment Evaluation Checklist (ATEC).	Improvement according to: mean CGI scores (p = 0.0008), receptive language (p < 0.0001), social interaction (p = 0.0473), and eye contact (p = 0.0102); ABC total score: no improvement except a greater number of children improved in irritability (p = 0.0311). ATEC: sensory/cognitive awareness improved (p = 0.0367) HBO: safe and well-tolerated.

[22]	Randomized, double-blind, placebo-controlled design	Children aged 2 to 14 (mean age = 6.18). 46 children enrolled in the study, 12 children withdrew, placebo group(n = 16 treatment group(n = 180	80 hourly sessions for both groups HBO group: 1.3 atm and supplemental oxygen (approximately 24-28% FiO2) placebo group: free airflow through the chamber at ambient pressure supplements, dietary modifications, and medical interventions were held constant during the intervention [mean number of Applied Behavior Analysis (ABA) treatment hours per month was 109 (HBO 114.7; placebo 103.3)]	Aberrant Behavior Checklist (ABC) Autism Diagnostic Observation Schedule-Generic (ADOS) Clinical Global Impression Scale (CGI) Direct observations	HOT is not more effective than placebo. 1 withdrew after a seizure attack.

In one of these studies conducted by Granpeesheh et al., both groups received eighty 1-hour sessions of HBO therapy (Table 2). The HBO therapy group received the compression to 1.3 atmospheric pressure with a supplemental oxygen (approximately 24-28% FiO2), while the control group received free airflow through the chamber at ambient pressure [21]. This study included the children aging from 2 to 14 who suffered from autistic disorder. In addition, participants could take any supplements, pharmacological interventions, and dietary modifications. Moreover, Applied Behavior Analysis (ABA) was administered for many patients in both groups. However, these interventions were constant during the study. Nevertheless, medical conditions, such as seizures, chronic or current sinus infections, or current otitis media, were considered as the exclusion criteria.

Overall, twelve out of the 46 patients withdrew from the trial. Therefore, only 16 patients in control group and 18 patients in the treatment group completed the trial. One of the withdrawals in the control group was due to the adverse effect; the patient had experienced seizure [21].

In addition to the direct observation, a large variety of assessments, such as the Aberrant Behavior Checklist [32], the Autism Diagnostic Observation Schedule-Generic (ADOS) [33], Behavior Rating Inventory of Executive Functioning [34], and Clinical Global Impression Scale (CGI), were administered, as well. The authors used intent-to-treat analyses to examine the results. However, only 34 out of 46 patients were included for data analysis. In fact, 12 patients who were excluded from the study were not included in the statistical analysis.

The primary outcome measures of social reciprocity, communicative approach, and repetitive behaviors were not different between the two groups after the interventions. Subtracting the pret-test and post-test scores of each subscales of the Social Responsiveness Scale (SRS) showed that the mean differences were not different between the two groups. In addition, the number of the patients in the two groups was not statistically different regarding the improved communication (3 versus 2) as well as socialization (3 versus 2) and the total score of ADOS (5 versus 4). Also, the results of the direct observation did not support the idea that HBO therapy was more effective than free airflow through the chamber at ambient pressure [21]. Overall, the authors concluded that HBO therapy with 24% oxygen at 1.3 atm did not treat the children with autism [21].

Another study, which was conducted by Rossignol et al., was a multicenter, randomized, double-blind, controlled trial including 62 children from 2 to 7 years old suffering from autism and had been randomly allocated in two groups [22]. Hyperbaric treatment group received 40 1-hour treatments of hyperbaric treatment at 1.3 atmosphere (atm) and 24% oxygen. The control group, on the other hand, received slightly pressurized room air at 1.03 atm and 21% oxygen. Seven patients (4 patients in the HBO therapy group and 3 patients in the control group) dropped from this study. Intent-to-treat analysis was used for statistical analysis and all the participants were included in the analysis [22]. The authors reported that HBO therapy improved the overall functioning (*p *= 0.0008), receptive language (*p *< 0.0001), social interaction (*p *= 0.0473), and eye contact (*p *= 0.0102). In addition, the total score of ABC and its subscale of irritability in the HBO therapy group were improved more than those of the control group [22].

### Nonrandomized studies

Bent et al. reported that HBO was effective for the treatment of autism, while it did not affect the cytokines level [17]. It needs to be noticed that none of the patients in that study had an abnormal level of cytokines at the beginning of the study [17]. As the authors mentioned, their study was an open-label one (Table 3). So, there is a speculation that this effect may be due to the placebo effects. Some symptoms may change in the clinical course of autism. Moreover, their participants were taking concomitant medications for the treatment of autism. Therefore, it cannot be guaranteed that the improvement is induced by the intervention or other effects. In addition, the parental bias in reporting the symptoms in an open label study needs to be considered, as well. Of course, the improvement measured by CGI was also reported by the study. Therefore, this difference cannot be explained by parental reporting bias.

**Table 3:** Summary of non-randomized trials

References	Methods	Participants	Interventions	Measurements	Outcome and adverse effects
[17]	Case series	10 children	40 days of HBO (1.5 atmosphere absolute; 100% oxygen) for 1 h, 5 days a week for 8 weeks, followed by a 4 week break, and then another 40 treatments over 8 weeks + Concurrent stable medical regime	Aberrant Behavior Checklist (ABC) Pervasive Developmental Disorder Behavior Inventory (PDDBI)	Significant improvements in total score and 3 of the 5 subscales of the Aberrant Behavior Checklist (ABC) including irritability, lethargy, and hyperactivity Improvements in 3 out of ten subscales of Developmental Disorder Behavior Inventory (PDDBI) No effects on cytokines level Adverse effects: 3 ear discomfort, 2 ear infections, 1 increased hyperactivity, 1 increased vocal sensitivity, 1 increased sensory needs, 1 insomnia, 1 dehydration, 1 fatigue, 1 irritability, 1 increased mouthing of objects, and 1 seizure.

[15]	An open-label pilot study	18 children with autism, 3 to16 years old	40 hyperbaric sessions of 45 minutes 1.3 atm and 24% oxygen or 1.5 atm and 100% oxygen Concurrent medication was allowed. However, no new medication was administered during the study.	Childhood Autism Rating Scale (CARS)	Significant improvements for motivation, speech, and cognitive awareness (p < 0.05). No major adverse events

[14]	Retrospective study	6 children with autism	40 sessions of one hour of HBO at 1.3 ATA an 28-30% oxygen + concurrent therapies	Autism Treatment Evaluation Checklist (ATEC), Childhood Autism Rating Scale (CARS), and Social Responsiveness Scale (SRS)	Decreased symptoms, well tolerated

[23]	Case series	7 children	HBO (1.3 Atmospheric pressure., 100% oxygen, 10 sessions) treatment	Used instrument is not mentioned	Improvement No serious adverse effect

[31]	Case series study	16 children with mean age of 5 years and 9 months (range = 3 years and 10 months-9 years and 5 months).	40 sessions of HBO (24% oxygen and 1.3 ATA) on 11 topographies of directly observed Behavior No change in medical treatment regimen or dietary regimen for 6 weeks prior to the study.	Direct observation Play sessions (a) vocal initiations behavior; (b) physical initiations; (c) vocal response; (d) physical response; (e) self-injurious behavior or aggression; (f) disruption; (g) tantrums; (h) vocal stereotypy; and (i) physical stereotypy	HBO neither improves nor worsens the autism symptoms.

[24]	Case report	Three children	88% (+/- 3%) oxygen at 1.3 ATA On topographies of behaviors	A Child Responses Measured was defined.	Two of the three children had improvements.

Another study which reported the efficacy of 40 hyperbaric sessions lasting for 45 minutes on autism was an open-label study [15]. According to the results, HBO therapy increased motivation, speech, and cognitive awareness. Moreover, there were no major adverse effects [15].

A case-series study reported that HBO is effective for treatment of the children with autism [25]. However, there are some ambiguities about its results; for instance, it is not clear whether the participants take any con-current medication. Moreover, they did not mention how they measured autism symptoms. They reported that 75% of patients improved; meanwhile, the criteria for the improvement are not described. However, that study has a strong point, it measured fine motor, eye-hand coordination, language development, and gross motor development. These items are more objective than social or communication problems.

A retrospective case study including 6 patients reported that HBO is effective. The average improvement in parent-reported Autism Treatment Evaluation Checklist (ATEC) ranged from 8.8% to 31.6% in older children and younger children, respectively. The results of the statistical analysis showed P value to be 0.0538 which is a trend for statistical significancy [14]. In addition, the average improvement on Childhood Autism Rating Scale (CARS) was 12.1% (*P *= 0.0178). Besides, the average score on Social Responsiveness Scale (SRS) was 22.1% with aP value of 0.0518 which again, shows a trend for a statistically significant difference. These results suggest promising effects. However, it is needed to be confirmed in further studies because these patients were allowed to take concomitant therapies and, at the same time, new therapies were allowed to be added during this retrospective case study [14].

### HBO therapy related adverse effects

In a study conducted by Granpeesheh et al., no barotrauma related adverse effects (such as pressure injury to tympanic membranes, and sinuses) were reported. Nevertheless, no adverse effect were reported for HBO therapy [21]. The other randomized controlled study also reported the HBO-related adverse effects including urinary frequency (1 case) and skin rash (1 case). Also, asthma symptoms were exacerbated in one patient in the treatment group (Table 2) [22].

## Discussion

There is only one controlled study supporting the efficacy of HBO therapy for autism. However, another randomized, double-blind, controlled trial did not support the efficacy of HBO therapy for the treatment of autism [21]. There are several points regarding that study [21]. First, 12 out of the 46 participants withdrew from the study. Therefore, the number of the patients in each group was limited; however, it is not clear whether the negative results can be attributed to the small sample size. In addition, the control group received free airflow through the chamber at ambient pressure. It is a debate whether this is really a placebo intervention. It should be noted that the participants in both groups showed improvement over time. Of course, it does not mean that other options for HBO therapy, such as other doses, are not effective. Furthermore, both groups were administered intensive ABA intervention during this study. One explanation for the lack of efficacy is that HBO therapy does not add significant therapeutic effects to ABA; therefore, It cannot be interpreted that HBO therapy is ineffective. However, it is not clear if direct observation has enough reliability and validity to be considered as an outcome measure.

The other randomized, double-blind, placebo-controlled trial reported that some children with autism can benefit from HBO therapy [22]. However, there are some concerns about that study. The measures used in that study were Clinical Global Impression scale, Aberrant Behavior Checklist (ABC), and Autism Treatment Evaluation Checklist (ATEC). In addition, an intention-to-treat approach was used for statistical analysis. One of the measures used in that study was CGI. It is expected that clinicians rate CGI to show the degree of changes. Although, CGI is not scored at baseline, both parents and physician scored CGI at baseline in that study. Moreover, the improvement of overall functioning, receptive language, social interaction, and eye contact were assessed according to CGI. Meanwhile, CGI is used in order to show the overall changes. Of course, the validity and reliability of CGI for assessment of overall functioning, receptive language, social interaction, and eye contact in autism should be investigated.

Of course, another scale was also used in that study. As the results showed, no difference was observed between the two groups regarding the ABC total score and subscale scores (*p *= ns). However, there was a trend for a significant difference between the two groups regarding the irritability subscale score (*p *= 0.0976).

Autism Treatment Evaluation Checklist (ATEC) Scale was also administered. Sensory/cognitive awareness in the treatment group improved more than that of control group. However, 10 patients in the treatment group and 8 patients in the control group were not assessed at baseline by this scale; therefore, the data for 44 patients were gathered. Nevertheless, it is not clear whether it has any impact on the results of this study. In addition, it is not clear whether the statistical differences are due to the alpha inflation.

There are some other possible explanations for the difference between these two studies' findings. a) The patients' diagnostic characteristics of these two trials are not similar. While one of them included the patients with autism spectrum disorders [21], the other study included the patients with autism [22]. In addition, the children with PDD-NOS, Asperger syndrome, and fragile X syndrome were excluded from the study [22]. b) One study supported the efficacy of HBO therapy according to CGI as an outcome measure [22], while the other study did not find these results using CGI. c) Only one of these two studies reported that the patients demographic characteristics (age and gender ratio) and baseline severity were not different between the treatment group and control group [22]. d) while one study included children aged 2 to 14 years old [21], the other study included those between 2 and 7 years old [22]. e) One study was a multicenter study [22], while the other study was conducted in one center [21]. It needs to be mentioned that the age of the children, autism severity, and the degree of improvement were not different between these six centers [22]. f) While the outcomes were assessed by the parents or primary caretakers and the treating physician [22], the trained assessors, who were blind to group assignment, and evaluated the outcome for another trial [22]. g) The assessment of blinding was conducted for one study [21], while it was not performed for the other one [21]. h) One study provided about 5 treatments per week [21], while the other study provided 10 treatments per week [22].

This systematic review has a limitation because only one author reviewed the articles and scored the quality of the experimental studies.

In conclusion, the results supporting the efficacy of HBO therapy are not replicated. In addition, none of these trials used placebo group. Therefore, these results are not conclusive for the efficacy of HBO therapy for the treatment of autism. However, the promising effects of case series studies and the only multicenter, randomized, controlled trial encourage conducting further clinical trials with more rigorous scientific methodologies. In general, since control group should receive some pressure to stimulate HBO therapy, it is not practical to conduct a placebo controlled study for HBO therapy. Therefore, sham controlled studies are recommended to be conducted. Examining different pressures and oxygen levels is suggested, as well. Further studies should consider that more than half of patients with pervasive developmental disorders suffer from attention deficit hyperactivity disorder as a co-morbidity [35].

Serious adverse effects are not reported in controlled studies. However, it does not guarantee that HBO therapy in higher pressures and oxygen levels is safe in autism. In fact, more studies including larger samples of patients are needed to be conducted.

## Competing interests

The author declares that they have no competing interests.

## Authors' contributions

The author has read and approved the final manuscript.
